# Persistent oral health disparity in 12-year-old Hispanics: a cross-sectional study

**DOI:** 10.1186/s12903-016-0162-7

**Published:** 2016-02-01

**Authors:** Augusto R. Elias-Boneta, Milagros J. Toro, Sona Rivas-Tumanyan, Margarita Murillo, Luis Orraca, Angeliz Encarnacion, Dana Cernigliaro, Carlos Toro-Vizcarrondo, Walter J. Psoter

**Affiliations:** School of Dental Medicine, Medical Sciences Campus, University of Puerto Rico, San Juan, Puerto Rico; Department of Dental Medicine, NYU-Lutheran Medical Center, Brooklyn, New York USA

**Keywords:** Dental caries, Children, Puerto Rico, Prevalence, Healthcare disparities

## Abstract

**Background:**

Dental caries is the most prevalent chronic illness worldwide. In the US dental caries has been described as a “silent epidemic”, affecting 58.2 % of 12–15 year-olds, particularly in minority and immigrant groups. Caries is associated with complex yet preventable biological and behavioral factors such as dental plaque and diet, as well as social determinants of health. In developed nations, a higher risk caries has been associated with populations of low socio-economic status (SES), especially in areas with greater income disparity. An island-wide study conducted in Puerto Rico in 1997 revealed a high prevalence of dental caries in 12-year-olds and a significant health disparity between children attending private and public schools. The purpose of the present study was twofold: 1) to estimate caries levels of 12-year-old school Puerto Ricans in 2011; and 2) compare results to data obtained in 1997 to explore any possible change in caries outcomes after a government health insurance (GHI) reform was implemented.

**Methods:**

In this cross-sectional study, a probability sample of 133 out of 1,843 schools was selected proportional to enrollment size, and stratified by 1997 GHI regions, school type, and gender. Calibrated examiners conducted oral soft tissue and caries examinations. Dental caries prevalence was estimated. Mean Decayed Missing Filled Tooth/Surface (DMFT/S) indices and mean Significant Caries Index (SiC) were calculated and compared retrospectively to data obtained in 1997.

**Results:**

The final sample included 1,587 school-enrolled children. About 53 % of participants were female and 77 % attended public schools. Between 1997 and 2011, reductions were observed in caries prevalence (81 to 69 %), mean DMFT scores (3.8 to 2.5), mean DMFS scores (6.5 to 3.9), and mean SiC index (7.3 to 5.6) in both private and public schools, with a more prominent decrease in private schools. Between 1997 and 2011, overall the filled component increased (50 to 67 %), while decayed and missing component decreased (42 to 30 %) and (8 to 3 %), respectively.

**Conclusions:**

Among 12-year-old schoolchildren in Puerto Rico between 1997 and 2011, caries prevalence, extent, and severity decreased as well as the DMFT missing component, while the filled component increased. Dental caries prevalence was high and the health disparity persists between children enrolled in public and private schools after more than a decade of the GHI implementation. The relationship between GHI implementation and other potentially relevant co-factors for caries warrants further research, as does the seemingly entrenched disparity across groups.

## Background

Dental caries is the most prevalent chronic illness worldwide. In the United States (US) it has been referred to as a “silent epidemic” affecting 58.2 % of 12–15 year-olds, particularly in minority and immigrant groups [1]. Caries are associated with complex, yet preventable, biological and behavioral factors such as plaque and diet [2–6], and social determinants of health [7], including structure and environment [8, 9]. Additionally, in developed countries, a higher risk of caries has been associated with populations of low socio-economic status (SES) [10] particularly in areas with large income disparities [11, 12].

Puerto Rico (PR), a non-incorporated territory of the US located in the Eastern Caribbean has a population of approximately 3,725,789 [13]. In 2011, PR had a poverty rate of 45.6 % [14] and high income inequality, measured by the Gini Index, of 0.531, compared to 0.475 in the mainland US [15]. In 1999, the poverty rate in PR was 48.2 % [16] whereas the Gini index was 0.564 [17].

The first island-wide study to estimate the dental caries prevalence in PR among school-enrolled 12-year-olds was conducted in 1997, where caries prevalence was found to be 81 % in a population of 1,435 children [18]. Further, the mean number of Decayed, Missing, and Filled Teeth (DMFT) and Surfaces (DMFS) were 3.8 and 6.5, respectively. The overall mean DMFS score reported was substantially higher than the mean DMFS reported for 12-to 17-year-olds in the United States [19] and also compared unfavorably with most of the other Caribbean countries at that time [20]. Findings from the 1997 study also identified oral health differences between children attending urban private schools (Prevalence = 75 %; mean DMFS = 4.7; mean DMFT = 3.0), rural private schools (Prevalence = 84 %; mean DMFS = 7.2; mean DMFT = 4.2) and urban public schools (Prevalence = 82 %; mean DMFS = 6.5; mean DMFT = 3.8), suggesting more localized oral health disparities.

From 1993 to 2000, the government of Puerto Rico implemented a Government Health Insurance (GHI) reform program affecting both medical and dental services [18], independent of the 1997 oral health study. The intention of the GHI was to provide third party health insurance to Medicaid and Medicare eligible [21] and the medically indigent (federal poverty level below 200 %) populations, to improve access, quality, and cost-effectiveness [22]. Prior to 1993, Puerto Ricans were entitled to health services offered in government-owned and -financed facilities; however, most patients were medically indigent [22]. Dental services were offered by centers for diagnosis and treatment (CDT) throughout the island, although they were limited to emergencies, extractions, and fillings (e.g., silver amalgam, esthetic resin, and temporary cement). Services such as dental sealants, pulpotomies, and stainless steel crowns (SSC) were not offered in CDTs at that time. After implementation of the health reform, in addition to the services provided prior to the GHI, limited preventive services (e.g., periodic dental evaluation with a complete radiographic examination every three years; bi-annual topical fluoride application for children under 19; dental sealants in temporary molars of children under eight with high risk for caries, and in permanent posterior teeth for children under 14), as well as pulpotomies and SSC are offered.

While health reforms have been implemented in various countries with the aim of improving health care and health outcomes, some that have included oral health components have had mixed and/or debatable outcomes [23, 24]. The 1997 PR study suggested that the partially initiated GHI reform may have been contributing to reducing the prevalence of dental caries, since prevention, treatment, and disease levels might be influenced by increased access to dental services [18]. However, there has been no assessment of the impact of GHI on oral health outcomes since its implementation, nor has there been a comprehensive, island-wide, dental caries survey in PR since 1997. A reassessment of the prevalence of dental caries and untreated disease after the implementation of the GHI program could provide useful information regarding dental caries trends in PR and the possible impact of the GHI on such trends.

The purpose of this study was twofold: 1) to estimate caries prevalence of 12-year-old school Puerto Ricans in 2011; and 2) compare results to data obtained in 1997 to explore any possible change in caries outcomes after a government health insurance (GHI) reform was implemented. This cross-sectional study and the study conducted in 1997 were initiatives of the University of Puerto Rico School of Dental Medicine and not conceived to serve as a surveillance activity.

## Methods

### Sampling frame

A multistage stratified sampling methodology was used for the accurate estimation of caries prevalence generalizable to the population of all 12-year-old Puerto Ricans enrolled in school during the study period. Existing estimates on the number of 12-year-olds residing in Puerto Rico differ, which the PR Department of Education (PRDE) attributes to an overestimation in the Puerto Rican population (18–33 %) by the US Census due to the timing of data collection (calendar year vs. school year) and the reduction of the Puerto Rican population in recent years [25]. The 2010 US Department of Commerce, Bureau of Census, estimated that there were 54,239 12-year-olds residing in PR [26]; however, the PRDE reported that approximately 46,574 12-year-olds were enrolled in the school system in 2010–11 [25]. The universe of public and private schools was used as the sampling frame for this island-wide cross-sectional epidemiologic study.

### Sample selection

**Fig. 1:**
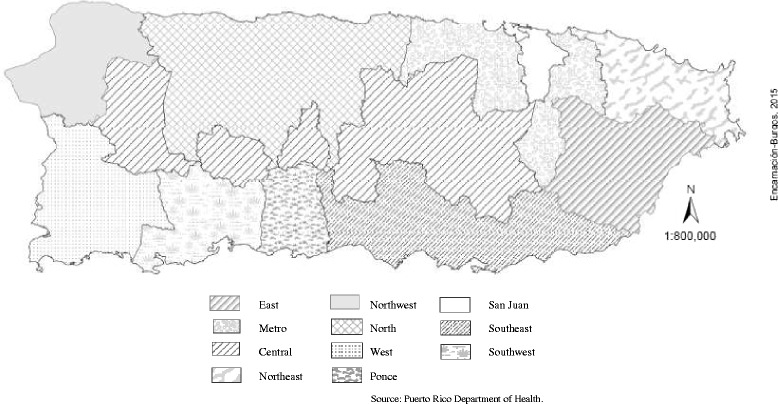
Map of Puerto Rico government health insurance regions, 1997

Prior to the 1997 study, the Puerto Rico Health Department divided the island into 11 administrative regions: “North”, “East”, “Metropolitan”, “San Juan”, “Central”, “Southeast”, “Ponce”, “Southwest”, “West”, “Northwest”, and “Northeast”. Figure 1 shows a map of the 11 GHI regions of PR. Although the number of health regions had been reduced by 2010, the 1997 distribution was employed in this study to allow caries data comparison. The list of all schools was stratified according to the GHI regions for administrative and operational purposes [18].

Public schools were additionally stratified according to the geographic zone (urban public and rural public). Within each stratum, schools were arranged according to their geographic proximity and poverty level. School level clustering reasonably assumed a local, social-cultural homogeneity at the school level in PR.

The number of public schools selected in each region was determined based on the distribution of 12-year-old public school students reported in each region, assuming a selection of a 20-student cluster in each school. The number of 12-year-olds in private schools was estimated based on the number of children enrolled between 5–7th grades in private schools and the proportion of 12 year-olds from 5–7th grade public schools. Public and private school enrollment has served as a surrogate for SES in a variety of studies globally [27–29]. Moreover, it has been reported that children attending public school have significantly higher caries experience [28] and treatment needs [29]. In PR, public/private school enrollment may be considered a proxy measure for the family’s socioeconomic status [18]. Furthermore, it has been stated that over 86 % of the students attending public school in PR are socio-economically disadvantaged [30]. This was confirmed in a subset sample of 122 children whose parents completed information on income [31].

The sample size of 1,500 subjects was targeted to estimate the national mean DMFS score, given the specifications of a 95 % confidence level, a maximum relative tolerable error of 7 %, a coefficient of variation equal to 1, and a clustering design effect of 1.2. In order to achieve the required final sample size, 133 schools (102 public and 31 private) were selected across the 11 regions, assuming 75 % eligibility and 75 % response rate.

### Recruitment

Permissions were obtained from the Department of Education and the school principals. The study was approved by the Medical Sciences Campus, University of Puerto Rico (MSC-UPR) Institutional Review Board (protocol # 0360105). A letter of invitation was sent to the parents of potential participants. Written parental/legal guardian consent and child assent, as well as medical history, and demographic information were obtained prior to enrollment in the study. Inclusion criteria for the study were 1) classification with a physical status ASA I and/or ASA II as defined by the American Society of Anaesthesiologist [32], and 2) being 12 years of age at the time of recruitment. Exclusion criteria included 1) participants with conditions requiring antibiotic prophylaxis or 2) those who demonstrated an inability to comply with study protocol requirements. In each of the selected schools, the list of 12-year-old students enrolled from 5 to 7th grades was requested from the school principal and/or the homeroom teachers, followed by the random selection of twenty 12-year-olds (10 boys and 10 girls) using a random number generator computer. A total of 1,587 subjects were evaluated from November 2010 through May 2011.

### Caries outcome variables

Caries experience, extent, and severity in this population were determined in terms of prevalence of dental caries (%), and by the estimated means of DMFT and DMFS scores. To identify possible dental caries disparities, the Significant Caries Index (SiC) [33] was also used. This index was introduced to bring attention to the individuals with the highest caries values in each

population, and determined by sorting children in the highest tertile according to their DMFT and calculating their mean score [33]. The SiC index helps identify individuals with higher dental needs and a greater potential public health impact in this group, especially when resources are restricted [34]. Diagnostic criteria for visual tactile determination were modified from the National Institute of Dental and Craniofacial Research (NIDCR), a branch of the US National Institutes of Health [35].

### Study procedures

#### Calibration

Prior to initiating the study, the examiners were trained and calibrated to a 0.75 level of agreement on the use of the modified NIDCR criteria [35]. During the study, repeated examinations were performed in 10 % of the subjects to assess inter- and intra-examiner reliability.

#### Exams

Two examiners performed the oral examinations utilizing portable equipment (dental chair, external light source, and air compressor) following the Occupational Safety and Health Administration (OSHA) infection control procedures. Caries were diagnosed using visual-tactile criteria, as in 1997, with a #23 explorer and a flat surface mirror. No radiographs were taken. Prior to the dental examination and under supervision, children brushed and flossed their teeth. During the dental examination, each tooth was air-dried to remove plaque/debris and to check for surface contour, color changes, minor cavitation, or sealants. The explorer was then used to remove any remaining plaque/debris and was gently placed on the tooth surface to prevent early lesion surface damage. Uncavitated carious lesions were not recorded. Missing and filled teeth were defined as missing or filled due to caries. The examiners confirmed their diagnosis with the child. Participants received an oral health status report. Data were recorded on a simplified NIDCR data entry form [18].

### Data management

All data was entered into Microsoft Excel spreadsheets and imported to SAS statistical software, version 9.3 (SAS Statistical Institute, Cary, NC), to be verified, managed, and analyzed.

### Analyses

All analyses accounted for sampling methods (sample was stratified by GHI regions and type of school, then clustered within schools) by specifying 1) strata, 2) cluster, and 3) assigning weights inversely proportional to the probability of selection into the sample, adjusted for non-response, and later normalized. As part of the descriptive analysis, weighted caries prevalence, mean DMFT/S, and mean SiC index were calculated with their respective standard errors and 95 % confidence intervals (95 % CIs). The same analysis was repeated while stratifying by region, gender, school type, and groups defined by the combination of gender and school type. We also calculated the percent distribution for each component of the DMFS index.

*P*-values for regional, gender, and school type differences were produced using multivariable regression models, taking into account the stratified cluster sample design. To obtain p-values for group differences in mean DMFS, DMFT, and SiC index, we employed Poisson [36] and negative binomial regression models; however, due to the similarity of the results obtained from the two regression methods, only p-values from the Poisson models were presented in the results. Caries prevalence differences between groups were studied using logistic regression. All models were adjusted for region, gender, and school type (private, public rural, public urban). To adjust for differences in analytic approach, data from the 1997 study was re-analyzed. The 1997 data did not include SiC index, used linear regression models for DMFS and DMFT and presented regression-adjusted means and prevalence estimates. For the present study, analysis was conducted by obtaining weighted (unadjusted) descriptive statistics for all measures and p-values from Poisson and logistic regression models. Differences between previously published and new estimates from 1997 were negligible.

## Results

Of the total 1,587 participants, 53.1 % were females, and 77.2 % were enrolled in public schools. The weighted Kappa statistics for DMFS and DMFT ranged from 0.84 to 1.0 for intra-examiner, and from 0.83 to 0.91 for inter-examiner reliability.

### Caries prevalence, DMFS, DMFT and SiC Index by GHI region

Table 1 provides caries prevalence, DMFS, DMFT and SiC Index for the study population by region. The overall caries prevalence was 69 % (95 % CI: 66, 73) ranging from 58 % in Ponce to 75 % in the Central region. The overall mean DMFS score for the study population was 3.9 (95 % CI: 3.5, 4.3), ranging from 3.2 in San Juan to 5.4 in the Central region. For DMFT scores, the average was 2.5 (95 % CI: 2.3, 2.8) and ranged from 2.0 in San Juan to 3.2 in the Central region.

**Table 1:** Caries prevalence, DMFS, DMFT, and SiC index^a^ in 12-year-old Puerto Ricans (2011) by GHI Regions^b^

					Caries prevalence			DMFS				DMFT				SiC index			
Region	Number	% N	Wt^c^ N	Wt^c^ N %	Percent	95 % CI		Mean	SE	95 % CI		Mean	SE	95 % CI		Mean	SE	95 % CI	
North	165	10.4	297	18.7	74	63	84	3.6	0.48	2.6	4.6	2.5	0.29	1.9	3.1	4.9	0.39	4.1	5.7
East	129	8.1	69	4.4	70	63	77	4.2	0.60	2.9	5.6	2.7	0.26	2.1	3.2	5.5	0.25	4.9	6.1
Metropolitan	363	22.9	581	36.6	67	60	74	3.5	0.33	2.8	4.2	2.4	0.23	1.9	2.8	5.0	0.25	4.5	5.5
San Juan	163	10.3	164	10.3	61	50	71	3.2	0.47	2.2	4.2	2.0	0.24	1.5	2.6	4.5	0.35	3.8	5.3
Central	223	14.1	243	15.3	75	65	84	5.3	0.57	4.1	6.5	3.2	0.27	2.6	3.8	5.8	0.18	5.5	6.2
Southeast	143	9.0	67	4.2	70	52	88	5.3	1.04	2.9	7.6	3.0	0.42	2.0	3.9	5.7	0.39	4.8	6.6
Ponce	68	4.3	29	1.8	58	41	76	3.8	0.48	2.6	5.0	2.4	0.26	1.8	3.1	5.8	0.36	4.9	6.8
Southwest	82	5.2	13	0.8	68	55	80	3.6	0.57	2.0	5.1	2.2	0.31	1.4	3.1	5.1	0.59	3.5	6.8
West	78	4.9	47	3.0	70	56	84	3.3	0.59	1.9	4.6	2.0	0.30	1.3	2.7	4.3	0.18	3.9	4.7
Northwest	77	4.9	46	2.9	72	58	87	4.2	0.43	3.2	5.2	2.7	0.21	2.2	3.2	5.0	0.23	4.5	5.5
Northeast	96	6.1	30	1.9	73	57	89	4.3	0.94	2.0	6.6	2.5	0.39	1.6	3.5	4.7	0.58	3.3	6.2
Total PR	1587	100	1587	100	69	66	73	3.9	0.20	3.5	4.3	2.5	0.12	2.3	2.8	5.6	0.12	5.4	5.9

^a^Weighted using normalized inverse probability weights

^b^Government health insurance regions, as in 1997

^c^Weighted

### Caries prevalence, DMFS, DMFT and SiC Index by demographic strata

Table 2 highlights oral health outcomes in the study population by demographic strata. Children attending private schools had a lower prevalence (55 %) relative to those attending public schools (72 % in both rural and urban public schools). No gender differences were observed. When gender and school type groups were analyzed, both females and males in public schools had a significantly higher caries prevalence (*p* < 0.05 for all comparisons) compared to males in private schools, the reference category with the lowest prevalence. Mean DMFS score was significantly higher among females compared to males after adjusting for covariates (4.2 vs. 3.5, *p* = <0.001). On average, children from public schools (both rural and urban) had a mean of 4.2 surfaces affected by caries (decayed, filled or missing due to caries), whereas private school attendees had approximately 2.5 surfaces involved (*p* < 0.001 for all comparisons after adjusting for gender and region). The highest mean DMFS was observed in female students enrolled in public rural schools, while private school-enrolled boys had the lowest mean DMFS (4.5 vs. 2.0, *p* < 0.001). The average decayed component of the DMFS index was 30 %; the average percentage of filled surfaces and missing due to caries surfaces was 67 and 3 %, respectively.

**Table 2:** Caries prevalence, DMFS, DMFT, and SiC index^a^ in 12-year-old Puerto Ricans by strata^b^ (2011)

Strata	n	Caries prevalence		DMFS			DMFT			SiC index		
		Percent	*p*-value^c^	Mean	SE	*p*-value^d^	Mean	SE	*p*-value^d^	Mean	SE	*p*-value^d^
Gender												
Female	842	71	0.29	4.2	0.25	<0.001	2.6	0.14	0.01	5.8	0.21	0.001
Male	745	68		3.5	0.23		2.4	0.14		5.0	0.13	
School type												
Rural public	597	72	0.03	4.2	0.33	<0.001	2.7	0.17	<0.001	5.5	0.13	<0.001
Urban public	629	72	0.001	4.2	0.29	<0.001	2.7	0.19	<0.001	5.7	0.22	<0.001
Private	361	55	REF	2.5	0.30	REF	1.7	0.16	REF	3.9	0.21	REF
Gender and school type												
Female, rural public	322	73	<0.01	4.5	0.40	<0.001	2.8	0.20	<0.001	5.5	0.18	<0.001
Male, rural public	275	71	0.02	4.0	0.37	<0.001	2.6	0.20	<0.001	5.6	0.22	<0.001
Female, urban public	338	73	<0.001	4.4	0.40	<0.001	2.8	0.23	<0.001	6.1	0.40	<0.001
Male, urban public	291	71	<0.01	3.9	0.34	<0.001	2.6	0.22	<0.001	5.4	0.19	<0.001
Female, private	182	60	0.06	2.9	0.44	<0.001	1.9	0.25	<0.001	4.2	0.36	<0.01
Males, private	179	50	REF	2.0	0.29	REF	1.4	0.17	REF	2.8	0.27	REF

^a^Unadjusted caries prevalence, DMFS, DMFT and SiC index estimates are weighted using normalized inverse probability weights

^b^Defined by gender, school type (3 groups) and the combination of school type and gender

^c^ *p*-values were obtained from multivariable logistic regression analysis, adjusting for gender, school type (3 groups) and the 11 health reform regions

^d^ *p*-values were obtained from multivariable Poisson regression analysis, adjusting for gender, school type (3 groups) and the 11 health reform regions

For DMFT outcomes, the mean DMFT of children enrolled in private schools (1.7) was statistically significantly lower compared to those enrolled in public rural schools (2.7; 95 % CI: 2.4, 3.0) and those in public urban schools (2.7; 95 % CI: 2.3, 3.1) (*p* < .001). Differences between the gender and school type groups were similar to those observed for the DMFS index.

The average SiC index for the study population was 5.6 (95 % CI: 5.4, 5.9). Significant differences (*p* = 0.001) were observed between the mean SiC for girls (5.8) and boys (5.0). Mean SiC index was also significantly higher in public schools (5.5 in rural and 5.7 in urban zone) compared to private schools (3.9) (*p* < 0.001 for both comparisons).

### Caries prevalence, DMFS, DMFT and SiC index comparisons from 1997 to 2011

**Fig. 2:**
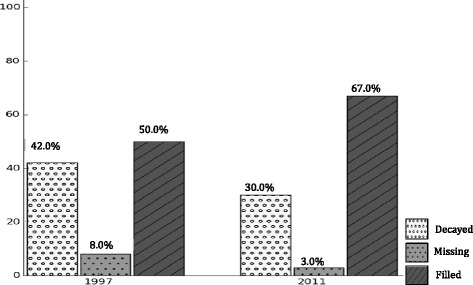
Decayed, missing, and filled components of the DMFS in 12-year-old Puerto Ricans (1997 and 2011)

As shown in Table 3, caries prevalence was lower in 2011 (69 %) compared to 1997 (81 %). Similar trends were observed for mean DMFS, DMFT, and SiC indices, as well. Gaps in caries distribution prevalence and extent measures between public and private school attendees increased over time and remained statistically significant in 2011. Children in 2011 had a higher percent (67 %) of the filled component of the mean DMFS, as opposed to 50 % in 1997. The share of the decayed and missing component of DMFS in 2011 was 30 and 3 %, respectively, compared to the 42 and 8 % reported in 1997 (Fig. 2).

**Table 3:** Caries prevalence, DMFS, DMFT, and SiC index^a^ in 12-year-old Puerto Ricans^b^ (1997 and 2011)

Year/	n	Caries prevalence		DMFS			DMFT			SiC		
School type												
		Percent	*p*-value^c^	Mean	SE	*p*-value^d^	Mean	SE	*p*-value^d^	Mean	SE	*p*-value^d^
1997												
Rural public	537	84	0.06	7.2	0.83	<0.001	4.2	0.37	<0.001	7.4	0.33	<0.001
Urban public	635	82	0.03	6.5	0.36	<0.001	3.8	0.18	<0.001	7.3	0.14	<0.001
Private	263	75	REF	4.7	0.56	REF	3.0	0.35	REF	5.8	0.26	REF
All	1,435	81		6.4	0.42		3.8	0.20		7.3	0.17	
2011												
Rural public	597	72	0.03	4.2	0.33	<0.001	2.7	0.17	<0.001	5.5	0.13	<0.001
Urban public	629	72	0.001	4.2	0.29	<0.001	2.7	0.19	<0.001	5.7	0.22	<0.001
Private	361	55	REF	2.5	0.30	REF	1.7	0.16	REF	3.9	0.21	REF
All	1,587	69		3.9	0.20		2.5	0.12		5.6	0.12	

^a^Unadjusted caries prevalence, DMFS, DMFT and SiC index estimates are weighted using normalized inverse probability weights

^b^Among all and by public-private school strata

^c^ *p*-values were obtained from multivariable logistic regression analysis, adjusting for gender and 11 health reform regions

^d^ *p*-values were obtained from multivariable Poisson regression models, adjusting for gender and 11 health reform regions

## Discussion

The purpose of this study was to estimate the prevalence of caries in 12-years-olds enrolled in school in Puerto Rico in 2011 and further explore changes in the distribution of dental caries between 1997 and 2011. The present study demonstrated a significant reduction in the prevalence of caries after the 1997 implementation of the GHI in PR, nevertheless findings also showed that dental caries remained high compared to the prevalence reported for US adolescents aged 12–15 in 2011–2012 [1]. The observed trends agree with most worldwide reports revealing a decrease in caries prevalence among children and young adults in the US, Japan, Sweden, Switzerland and Netherlands [37–40]. The mean DMFT in the present study is higher than that reported for 12-year-olds in the US during 1999–2004 [41], and slightly higher than values for 12-year-olds in the Caribbean and the Americas [42]. Interestingly, most Caribbean nations with community fluoridation programs have lower DMFTs compared to those without fluoridation [42]. Unfortunately, most published data in the region is outdated, thereby preventing appropriate data comparisons.

A significant oral health disparity in dental caries persists between 12 year-old students enrolled in public vs. private schools, as well as between male and female students. A comparison of the caries data between 1997 and 2011 shows a larger reduction in prevalence among children enrolled in private school as compared to those in public school (both rural and urban) (Table 3). Results for mean SiC index show a reduction between 1997 and 2011; however, this value is far from the global goal of a mean SiC of <3 for 12-year-olds by 2015 [33], and more than double the value reported for children of the same age in other countries [29, 43]. Moreover, the comparison of mean SiC index data from 1997 and 2011 revealed a larger reduction in caries severity among children enrolled in private schools as compared to public schools, confirming this avoidable and unjust inequity. Our findings agree with a previous study conducted in Nevada (USA) which reported higher mean SiC index for females and 13 to 15 year olds compared to 16 to 19 year olds (both genders); however, the overall mean SiC score was higher than in our study possibly due to the inclusion of older participants (13–19 years) in their cohort [34].

Since the intent of the PR GHI reform was to improve access and quality of health services for the medically indigent population, we anticipated a decrease in the dental caries gap between public and private school children between 1997 and 2011 [18]. However, an increased gap was observed after the GHI implementation and a greater difference in mean DMFT between public rural schools and private schools was observed for 2011 compared to 1997. On the other hand, an increase was observed in the filled component of the mean DMFS along with a decrease in the decayed and missing components, suggesting an increase in dental access. While increased access to dental care provided by the GHI reform may have contributed to a decline in dental caries among 12-year-old school children in PR, a report evaluating the PR health system, indicated that 48 % of GHI beneficiaries did not use their access to dental services [44]. Other factors, such as fluoride exposure from a variety of sources [3], consumption of dietary sugar [45, 46], and social determinants of health [9, 47] among others, may similarly influence the development and control of dental caries.

Fluoride availability seems to have played a major role in the marked decline of caries prevalence in some countries [3], even without access to better dental services or improved oral hygiene habits [48]. By 1960, 70 % of PR communities received fluoridated drinking water [49] and while this service was interrupted in the late 80s due to budget constraints [50], fluoridation was then government mandated in 1988 [51]. However, as of November 2015, fluoridation has yet to be implemented. Recently, the U.S Department of Health and Human Services recommended a fluoride concentration in drinking water of 0.7 parts per million (ppm) [52]. Throughout the island, drinking water fluoride levels have ranged from 0.063 to 0.123 parts per million (ppm) from 2005 to 2011 [53].

Recent analyses suggest that limiting sugars to less than 5 % of energy intake (E) may diminish the risk of dental caries through the life-span [54], and that sugar consumption between (<3 % E - < 5 % E), or even lower (2–3 % E) [38], are ideal and should be recommended, regardless of fluoridation availability [47]. A secondary analysis of our study population revealed that children with high caries prevalence consume significantly higher amounts of total sugars, fructose and inositol, especially in beverages throughout the day (results not shown).

Populations of low socioeconomic status (SES) are disproportionately affected by oral health diseases [9] even in countries with well-developed dental health care systems and community water fluoridation programs [48]. Additional research using a social determinants of health framework may further explain the health disparities seen between public and private school children in PR, as this disparity may be attributable to concurrent factors such as increased obesity, SES, access to care, and geographical distribution of dental services [55], in addition to effects of the GHI reform implementation. Regarding SES, the 2010 Census did not assess socio-economic data for the PR population [21], and the only available data that could be used to assess poverty trends derives from community surveys with large marginal errors due to the small sample size. It is necessary to obtain good quality data in order to make comparisons within PR and between PR and other nations. Future studies including socio-economic information, access to dental services, and the geographical distribution of such services, may be helpful to better understand the uneven reductions of dental caries and other secular changes. To address residual oral health disparities in 12-year-old Puerto Rican children, effective evidence-based public health interventions, such as water fluoridation [56], pit and fissure sealants [57], and fluoride varnish [6] programs, as well as the elaboration of a panoptic prevention policy with sugar intake reduction are recommended. In addition, research to impact care utilization targeting a comprehensive approach to preventive care [58] and follow-up studies to evaluate preventive strategies and oral health status are needed.

The study design and probabilistic stratified sampling methodology were employed to obtain nationally representative estimates of dental caries prevalence for Puerto Rico; however, the study was not designed to detect regional differences in those estimates. Since the number of school children included in some regions was small, we refrained from over-interpreting regional estimates. However, it is important to highlight that findings regarding caries trends between regions are consistent in both cohorts (1997 and 2011). The prevalence of caries was consistently higher in the Central region, whereas the lowest caries prevalence remained in the Ponce region [18]. The greatest reduction in caries prevalence between 1997 [18] and 2011 was observed in the San Juan region, whereas no reduction was observed for the Northern region. The highest mean DMFT and mean DMFS were also observed in the Central region for both cohorts (1997 and 2011). The greatest reductions in mean DMFT index, when comparing results from 1997 [18] and the present 2011 data, were observed in the San Juan, Northeast, and Central regions, whereas a modest reduction was observed in the North region. With reference to the mean DMFS, the greatest reductions were observed in the Northeast, San Juan, and Northwest while a relatively small reduction was observed in the Southeast region. As mentioned earlier, additional investigation is needed to gain insights on dental caries prevalence and severity within regions, taking into account differences in socioeconomic, social structure, environment, behavioral, and/or biological factors. The present study is the phase 1 (detecting phase) of a conceptual framework to advance our oral health disparity research agenda. Further research to understand (phase 2) and reduce (phase 3) oral health disparities in this population is essential [59]. The present publication did not address individual-level information about other risk factors, such as sugar intake, dietary patterns, knowledge, attitudes, tooth brushing, and parental educations, among others. Future publications will address the effect of these factors on dental caries.

There are several limitations to our study: the population examined was limited to 12-year-olds. The reasons for studying this age group were twofold. First, in 1997 WHO established 12 years as the global monitoring age for international comparisons [60], and secondly to allow comparisons with the 1997 cohort. Longitudinal studies in different age groups, using a broader sample size within regions, could evaluate caries trends in PR and help establish appropriate public health strategies. To allow caries data comparison, uncavitated lesions were not included in the study, as in 1997. We recognize that this is a weakness of our study since the exclusion of early caries lesions may underestimate the caries prevalence in this population. Another study limitation may be the lack of individual level economic data, and the use of a surrogate socioeconomic measure by school type, which may introduce misclassification. However, this misclassification is expected to be non-differential and most likely be introducing a small amount of bias towards the null hypothesis (no differences between high and low SES group).

At the same time, the current study has notable strengths. Probability sampling from all regions of PR allowed valid assessment of dental caries prevalence in this age group, even though it was not designed to produce region-specific prevalence estimates. The use of the SiC index allowed us to confirm disparities in dental caries. Overall, the study methods were similar to those employed in the 1997 study allowing caries data comparison. Moreover, dental examinations were conducted, in 1997 and 2011, by dentists trained by the same reference examiner. This allowed high intra- and inter-examiner reliability and will allow data comparisons with other studies locally and worldwide.

## Conclusion

Among 12-year-old schoolchildren in Puerto Rico between 1997 and 2011, caries prevalence, extent, and severity decreased as well as the DMFT missing component, while the filled component increased. Dental caries prevalence was high and the health disparity identified in 1997 persists between children enrolled in public and private schools after more than a decade of the GHI implementation. The relationship between GHI implementation and other potentially relevant co-factors for caries warrants further research, as does the seemingly entrenched disparity across groups.

## Abbreviations

ASAI/ASAII: American Society of Anaesthesiologist I/II
CDT: Centers for Diagnosis and Treatment
DMFS: Decayed Missing Filled Surface
DMFT: Decayed Missing Filled Tooth
DMFT/S: Decayed Missing Filled Tooth/Surface
E: energy intake
GHI: government health insurance
MSC-UPR: Medical Sciences Campus, University of Puerto Rico
NIDCR: National Institute of Dental and Craniofacial Research
OSHA: Occupational Safety and Health Administration
ppm: parts per million
PR: Puerto Rico
PRDE: Puerto Rico Department of Education
SES: socio-economic status
SiC: Significant Caries Index
SSC: stainless steel crowns
US: United States
